# Research Progress on Laser Additive Manufacturing of Oxide Dispersion-Strengthened Alloys—A Review

**DOI:** 10.3390/ma18174094

**Published:** 2025-09-01

**Authors:** Qian Zheng, Yan Yin, Chao Lu, Xiaoli Cui, Yutong Gao, Heng Zhu, Zhong Li, Junwei Shi, Wenqing Shi, Di Tie

**Affiliations:** 1 School of Materials Science and Engineering, Guangdong Ocean University, Yangjiang 529500, China; 2 School of Materials Science and Engineering, Lanzhou University of Technology, Lanzhou 730050, China

**Keywords:** additive manufacturing, oxide dispersion-strengthened, powder bed fusion, direct energy deposition, wire arc additive manufacturing

## Abstract

Oxide dispersion-strengthened (ODS) alloys are regarded as one of the most promising materials for Generation IV nuclear fission systems, owing to their exceptional attributes such as high strength, corrosion resistance, and irradiation tolerance. The traditional methods for fabricating oxide dispersion-strengthened (ODS) alloys are both time-consuming and costly. In contrast, additive manufacturing (AM) technologies enable precise control over material composition and geometric structure at the nanoscale, thereby enhancing the mechanical properties of components while reducing their weight. This novel approach offers significant advantages over conventional techniques, including reduced production costs, improved manufacturing efficiency, and more uniform distribution of oxide nanoparticles. This review begins by summarizing the state of the art in Fe-based and Ni-based ODS alloys fabricated via traditional routes. Subsequently, it examines recent progress in the AM of ODS alloys, including Fe-based, Ni-based, high-entropy alloys, and medium-entropy alloys, using powder bed fusion (PBF), directed energy deposition (DED), and wire arc additive manufacturing (WAAM). The microstructural characteristics, including oxide particle distribution, grain morphology, and alloy properties, are discussed in the context of different AM processes. Finally, critical challenges and future research directions for laser-based AM of ODS alloys are highlighted.

## 1. Introduction

With the rapid development of the economy, traditional fossil energy sources can no longer meet people’s needs due to over-exploitation and high carbon dioxide emissions. Consequently, the attention has begun to shift to the exploration of alternative energy sources, including wind, solar, geothermal, nuclear, tidal, and others [1,2]. In particular, nuclear energy has emerged as a major contender for solving the energy crisis owing to its high efficiency and cleanliness. Nevertheless, the intricate internal workings of the nuclear reactor necessitate that the cladding tube materials exhibit not only high resistance to neutron irradiation and commendable corrosion resistance but also optimal mechanical properties and resilience to creep at elevated temperatures [3,4]. Oxide dispersion-strengthened (ODS) alloys are regarded as the most prospective materials for Generation IV nuclear fission energy systems, given their exceptional oxidation resistance, irradiation resistance, creep resistance, corrosion resistance, and commendable mechanical properties at elevated temperatures [5,6]. Despite the considerable potential of ODS alloys for use as pipe cladding materials, technical difficulties in large-scale preparation remain significant obstacles to their wider application. In addition, traditional preparation techniques are extremely challenging for complex parts, which restricts the industrial application of ODS alloys. In recent years, the rapid development of additive manufacturing (AM) technology is expected to solve this problem.

**Figure 1:**
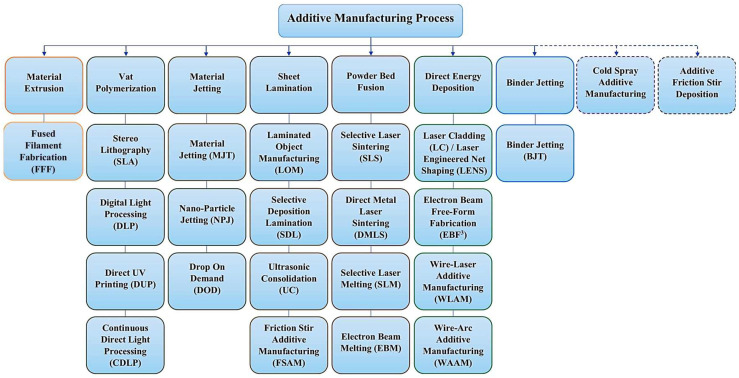
Classification of additive manufacturing [10].

**Figure 2:**
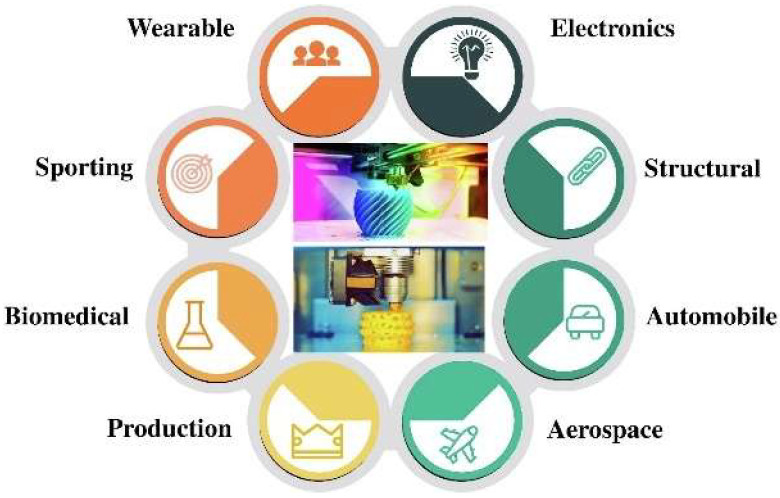
The application diagram of the AM process [14].

Conventional manufacturing techniques, such as casting and forging, involve the removal or reduction of raw materials. In contrast, AM, a novel non-contact manufacturing process which accumulates raw materials layer by layer. The term “additive manufacturing” is also used interchangeably with “3D printing” or “rapid prototyping technology”. As shown in Table 1, AM technology features the advantages of high manufacturing efficiency, low manufacturing cost, and high part complexity [7,8]. At present, AM technology can be divided into seven categories: powder bed fusion, direct energy deposition, and so on (Figure 1) [9,10]. AM is employed in a multitude of fields, including medicine, aerospace, construction, and others [11,12,13]. The application diagram of the AM process is shown in Figure 2 [14].

## 2. Introduction of ODS Alloys

**Figure 3:**
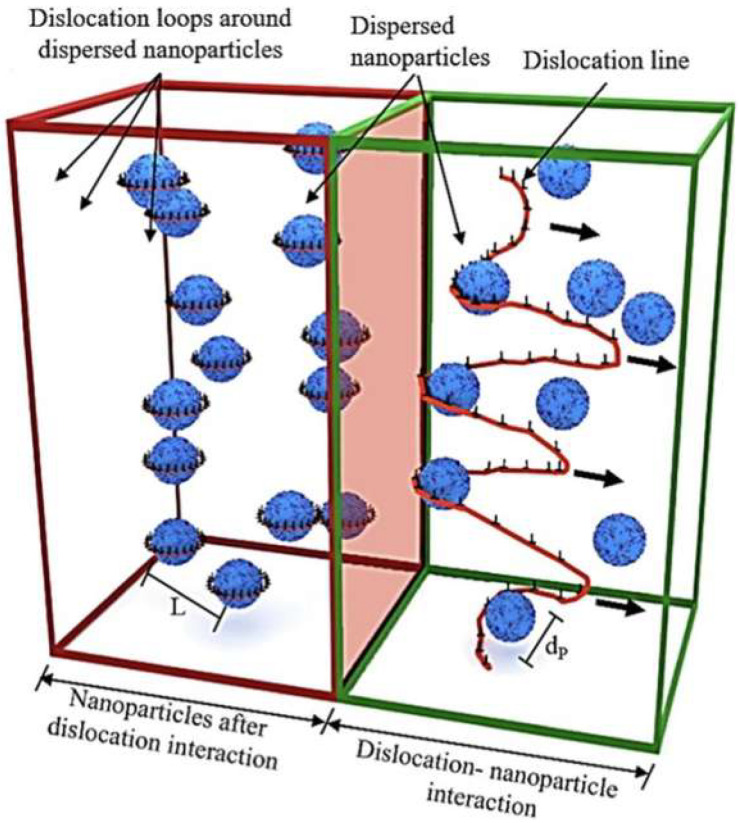
Schematic depiction of the Orowan mechanism in ODS alloys [15].

The term ‘ODS alloys’ refers to the incorporation of nanoscale oxide particles into the metal matrix [15]. The dispersion of the second phase results in the strengthening of the material through several mechanisms, including solid solution strengthening, fine grain strengthening, dispersion strengthening, and dislocation strengthening. The primary strengthening mechanism is the Orowan mechanism, whereby dislocations are unable to cut through the peroxide particles, resulting in bypass behavior. Figure 3 illustrates the Orowan mechanism diagram of the interaction between oxide particles and dislocation lines, both during (green frame) and after (red frame) the process [15]. The ODS alloys exhibits excellent mechanical properties, radiation resistance, and creep properties at high temperatures [16,17,18].

Yttrium-based ODS alloys are widely used in the nuclear industry, fuel generation, and other high-temperature environments because yttrium oxide particles significantly reduce the expansion of cavities under neutron irradiation [19]. Regarding the metal matrix, high-temperature iron-based alloys and high-temperature nickel-based alloys are the most studied.

### 2.1. Iron-Based Alloys

**Figure 4:**
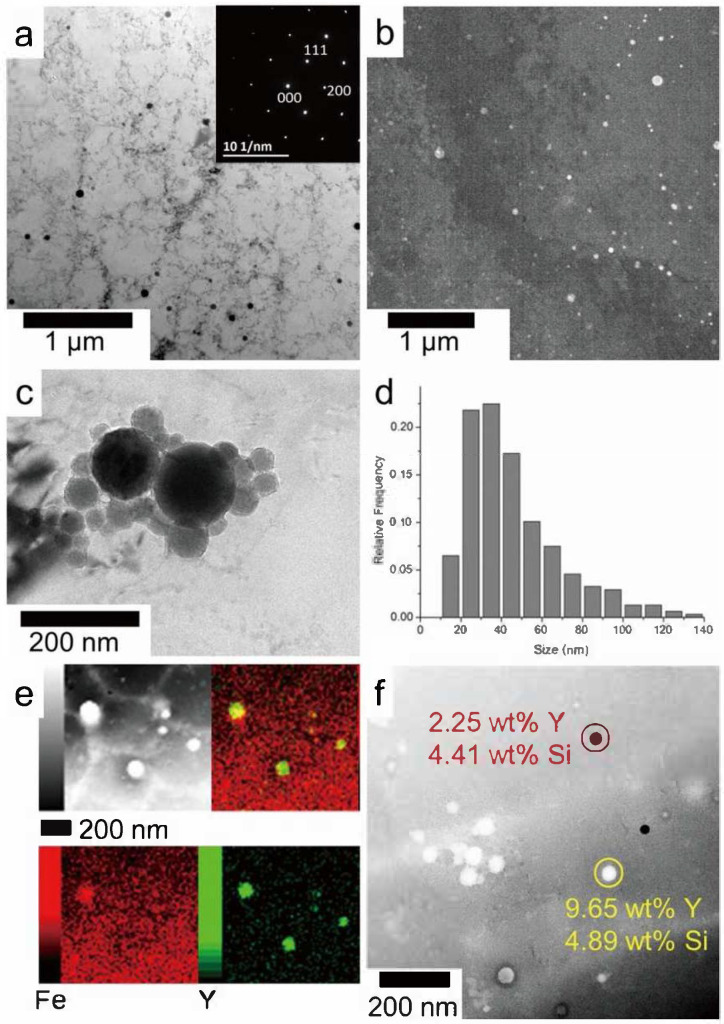
TEM characterization of the nanoinclusions in the sample 1 wt.% Y_2_O_3_-316L: BFTEM (**a**) and DFTEM (**b**) images, the selected area diffraction pattern was inserted in (**a**); BFTEM image showing agglomeration of nanoinclusions (**c**); calculated size distribution of oxide inclusions (**d**), TEM EDS mapping on several large nanoinclusions (**e**); EDS results on two nanoinclusions with different compositions (**f**) [26].

**Figure 5:**
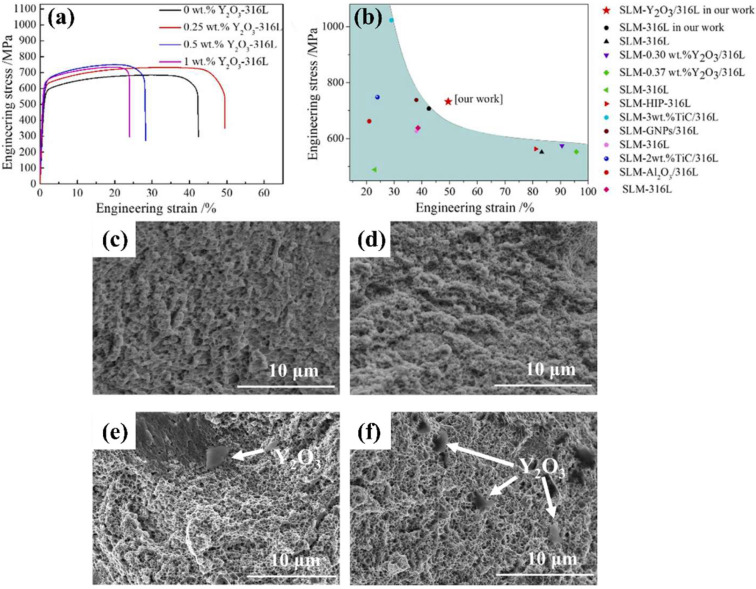
Tensile results: (**a**) the tensile stress–strain curves of as-printed specimens along the horizontal direction with different Y_2_O_3_ contents, (**b**) the comparison of the tensile properties of the related 316L specimens, (**c**) tensile fracture morphology of the 0 wt.% Y_2_O_3_-316L specimen, (**d**) tensile fracture morphology of the 0.25 wt.% Y_2_O_3_-316L specimen, (**e**) tensile fracture morphology of the 0.5 wt.% Y_2_O_3_-316L specimen, and (**f**) tensile fracture morphology of the 1 wt.% Y_2_O_3_-316L specimen [27].

**Figure 6:**
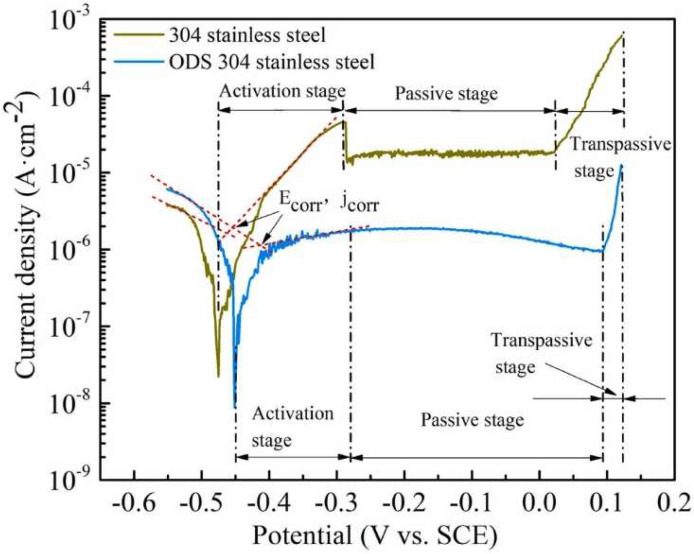
The potentiodynamic polarization curves of 304 alloys and ODS 304 alloys [28].

Ferritic ODS alloys exhibit excellent high-temperature creep properties, high-temperature oxidation resistance, and irradiation resistance [20]. However, the fracture properties of ferritic ODS alloys are poor due to the anisotropy. Additionally, the thermal embrittlement sensitivity of high Cr ferrite ODS alloys limits their application [21,22,23]. Parida et al. [24] demonstrated the formation of Y-Ti-O complex oxides promoted by addition of Ti to ODS alloys. The study showed that ODS steel exhibited superior creep fracture life compared to modified P91 (9Cr-1Mo) steel. Wang et al. [25] prepared 9Cr-3Al-ODS alloys and demonstrated that the addition of Al can reduce the precipitation of coarse M23C6 and optimize the size of carbides in ODS steel. The formation of large-sized Y-Al-O nanoparticles was observed following Al addition, which resulted in a reduction in the strength of ODS steel but an improvement in its ductility. Zhong et al. [26] found that nano-oxide particles rich in Y and Si with sizes ranging from 10 to 70 nm were uniformly distributed in the 1 wt.% Y_2_O_3_-316L matrix (Figure 4). During deformation, these nano-oxide particles can pin dislocations, enabling the 1 wt.% Y_2_O_3_-316L alloys to achieve a yield strength of 574 MPa and an elongation at break of 90.5%, both of which are superior to those of the 316L alloys. Hu et al. [27] prepared ODS 316L alloys with in situ chemically synthesized 0.25 wt.% Y_2_O_3_ via LPBF. As shown in Figure 5a,b, the study revealed that compared with other 316L specimens, the ODS 316 alloys not only exhibited excellent mechanical properties (with a strength of 732 ± 5 MPa) but also showed significantly improved plasticity (49.5 ± 0.8%), thus achieving an optimal combination of strength and plasticity. The enhancement in strength can be attributed to the hindrance effect of stacking faults and twin boundaries in the ODS 316L alloys on dislocation movement, while the improvement in plasticity is due to the significant increase in the number of dimples in the alloys caused by the addition of Y_2_O_3_ (Figure 5c,d). However, when the content of yttrium oxide exceeds 0.5 wt.%, agglomeration occurs, and these agglomerated regions are prone to crack propagation and fracture (Figure 5e,f). Zhang et al. [28] compared the corrosion resistance of ODS-304 alloys with that of forged 304 alloys, and the results are presented in Figure 6. The polarization curves of the two alloys are similar, and both can be divided into three regions: the active region, passive region, and transpassive region. However, due to the finer grain size of ODS-304 alloys (6 μm) compared to 304 alloys (56 μm), ODS-304 alloys exhibit a higher self-corrosion potential (−416 mV), a smaller self-corrosion current density (113 μA/cm^2^), a higher open-circuit potential (−279 mV), and a higher pitting potential (90 mV). These characteristics result in ODS-304 alloys having superior corrosion resistance to 304 alloys. Xu et al. [29] found that Si doping increased the diffusion rate of Cr and Mn in Si9Cr-ODS alloys. Consequently, the incorporation of Si is advantageous for enhancing the oxidation resistance of ODS steel. Getto et al. [30] observed the coarsening of Y-Al-O dispersions by irradiating ODS-MA956 after friction stir welding with 5 MeV Fe ions at 450 °C. Leojro et al. [4] performed a heat treatment of ODS-MA956 at 1285 °C and found that Y-Al-O grew from an average of 34 nm to 41 nm, which may lead to a failure of the oxide’s ability to trap defects and pinned dislocations. Yaocf et al. [31] performed small punch tests on ODS-MA956 irradiated at a dose of 18.8 dpa, which revealed that the helium embrittlement of the ODS-MA956 alloys increased almost linearly with the increasing irradiation dose.

### 2.2. Nickel-Based Alloys

Nickel-based alloys demonstrate superior overall performance at elevated temperatures in comparison to conventional materials. It is a common component in fossil fuel plants and aerospace applications [32]. He et al. [33] demonstrated that the strength of nickel-based ODS alloys is primarily influenced by dislocation strengthening, precipitation strengthening, and grain boundary strengthening. In a study by Li et al. [34] produced a Y_2_O_3_ dispersion-strengthened nickel-base alloy (Y_2_O_3_-NiMo) by powder metallurgy. It was demonstrated that the uniform dispersion of Y_2_O_3_ markedly enhanced the mechanical properties of the material. Furthermore, the Y_2_O_3_-NiMo alloy exhibits excellent resistance to swelling and corrosion by molten salt. Gaikwad et al. [35] prepared a nickel-based ODS alloy (Ni-20Cr-20Fe-0.6Y_2_O_3_) doped with Y_2_O_3_ particles by powder metallurgy. It was found that 25.6 nm Y_2_O_3_ and YCrO_3_ particles were uniformly distributed inside the grains and at the grain boundaries of Ni-20Cr-20Fe-0.6 Y_2_O_3_. The yield strengths were 732 MPa and 497.82 MPa at 25 °C and 650 °C. However, at 750 °C, due to the easier slip of nanoparticles under external forces, the Orowan strengthening effect is weakened, and the yield strength of the ODS alloy drops to 259.12 MPa.

## 3. Preparation Technology of ODS Alloys

### 3.1. Traditional Preparation Methods of ODS Alloys

At present, the main methods for preparing ODS alloys are internal oxidation and mechanical alloying, which mainly include the following steps: the preparation of dispersion-strengthened alloy powder, solidification of alloy powder, mechanical processing, and heat treatment [15,24,36].

#### 3.1.1. Internal Oxidation Method

The internal oxidation method is a process whereby solute elements are oxidized to form corresponding oxide particles in the preparation of alloy powder [36]. The method has the advantages of low cost, high material utilization, and low environmental pollution. Nevertheless, the powder produced by the internal oxidation method is coarser and more unevenly distributed.

Schneibel et al. [37] produced ODS steels containing Y_2_O_3_, YFeO_3_, Y_2_Ti_2_O_7_, and Fe_2_TiO_4_ by the internal oxidation of Fe_17_Y_2_ and Fe_11_YTi intermetallic compounds under a mixture of Fe and Fe_2_O_3_ as an oxygen partial pressure. The hardness of the resulting material is comparable to that of the original intermetallic compounds. Upon annealing at 1000 °C, the microstructure of the alloy exhibits a more pronounced coarsening than that of the ODS alloys produced by mechanical alloying. Zhao et al. [38] prepared oxide dispersion-strengthened copper alloys (ODS-Cu) using titanium-doped copper powder as the raw material via a combined process of powder metallurgy and internal oxidation. The researchers compared the mechanical properties of these ODS-Cu specimens with those of Glidcop-25 (a commercial dispersion-strengthened copper alloy without titanium doping). It was found that the microhardness, yield strength, and tensile strength of the titanium-doped ODS-Cu specimens were 151.5 HV 0.1, 385.6 MPa, and 471 MPa, respectively—all higher than the corresponding properties of Glidcop-25 (microhardness: 118 HV 0.1; yield strength: 331 MPa; tensile strength: 421 MPa).

#### 3.1.2. Mechanical Alloying (MA)

**Figure 7:**
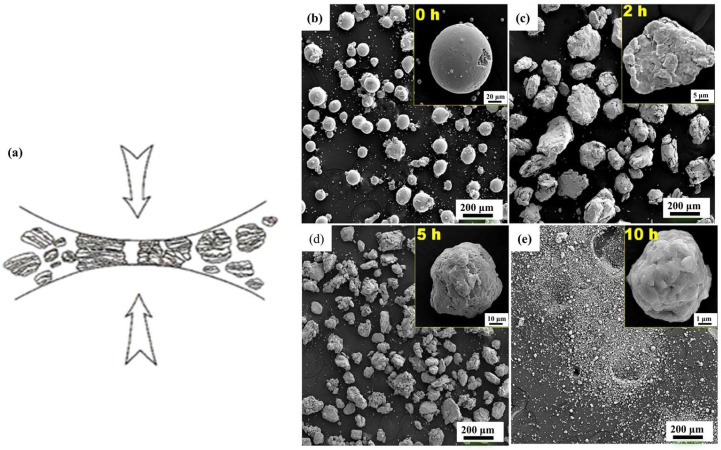
(**a**) Collision between grinding ball and alloy powder during mechanical ball milling [39]; mechanical grinding time of ODS alloys powder: (**b**) 0 h, (**c**) 2 h, (**d**) 5 h, (**e**) 10 h [24].

The MA method is also known as the dry powder high-energy ball milling method. The mixture of steel powder and Y_2_O_3_ powder is typically placed in a high-energy ball mill, where the grinding ball and the mixed powder are repeatedly impacted. As shown in Figure 7a, intense collisions occur between the grinding balls and alloy powder during the ball milling process [39].

The ball milling process involves the collision of grinding balls and powder. Figure 7b–e illustrates the morphology of the 9Cr ODS alloys powder under different grinding times. At the beginning of ball milling, the average particle size of the coarse powder and fine powder was observed to be 120 μm and 4.11 μm, respectively in Figure 7b. At the 2 h mark, the powder particles underwent extrusion and cold welding, resulting in an average particle size of 170 μm (Figure 7c). As the grinding process continued, the powder particles underwent gradual refinement, reaching an average size of 50–150 μm at the 5 h mark (Figure 7d). At the 10 h mark, the powder exhibits characteristics of spheroidization, with a particle size range of 1–10 μm (Figure 7e) [24]. Meanwhile, severe deformation can result in the generation of many dislocations, which repeatedly split the broken oxide. There is a risk that harmful phases may be generated in the collision process due to contamination of the powder during longer processing times [40]. Furthermore, during the high-temperature annealing process, the mechanical alloy material will form pores, which will significantly diminish the mechanical properties of the material [41]. Zhao et al. [42] used Fe-Cr-W-Y pre-alloyed powder and TiO_2_ powder, high-energy ball milling for 20 h. Fine-grained 13Cr-1W steel with an average grain size of 626.75 nm was prepared by Spark Plasma Sintering (SPS) at 950 °C and 80 MPa. Following annealing, ultrafine oxide dispersions with an average size of 11.15 nm were observed to be uniformly distributed on the substrate. The combined effect of precipitation strengthening, grain boundary strengthening, and dislocation strengthening results in yield strength and ultimate tensile strength of the alloy reaching 1352.82 MPa and 1559.60 MPa, respectively. Moreover, the tensile properties of the alloy are superior to those of the majority of commercial ODS steels.

**Figure 8:**
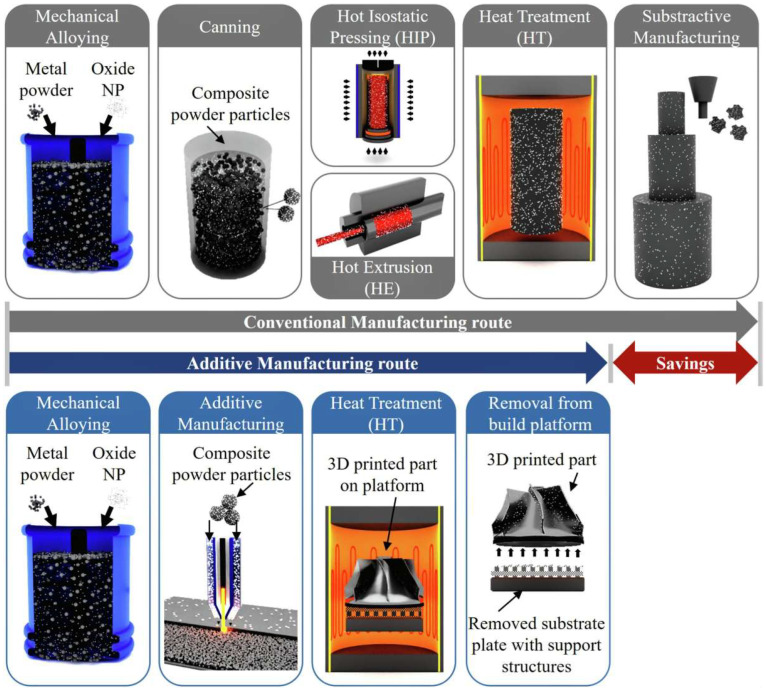
Comparison of conventional manufacturing route and additive manufacturing route of ODS alloys [15].

As shown in Figure 8, the conventional manufacturing route for ODS alloys involves complex processes such as MA composite powder manufacturing, hot isostatic pressing and hot extrusion powder metallurgical solidification, and heat treatment. AM technology involves controlling the composition and geometric configuration of materials below the nanoscale, thereby minimizing the weight of the material while improving the mechanical properties of part [43]. Compared with traditional ODS alloys preparation methods, AM has the advantages of low production cost, high efficiency, and a uniform distribution of oxide particles. Moreover, it can realize the near-net shaping of ODS alloys components with complex structures, thus avoiding subsequent machining processes (Figure 8).

### 3.2. The Potential of Additive Manufacturing ODS Alloys

Nowadays, the most commonly employed metal AM techniques include powder bed fusion (PBF), direct energy deposition (DED), and wire and arc additive manufacturing (WAAM) [44].

**Figure 9:**
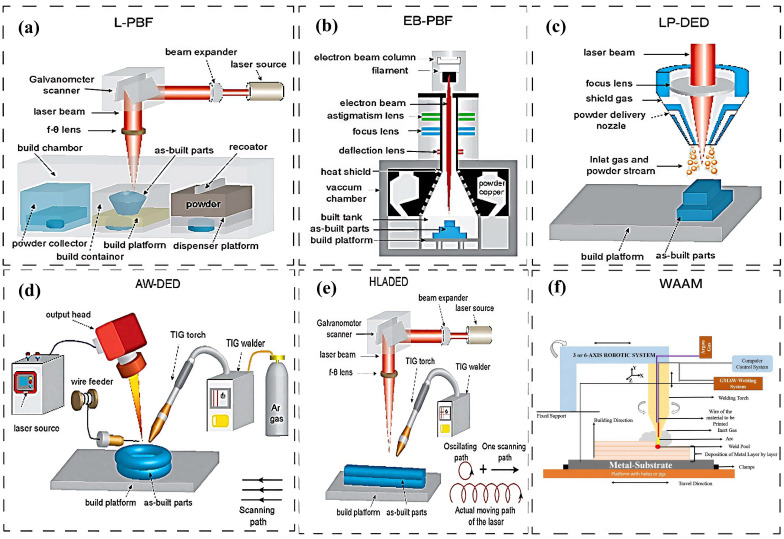
Additive manufacturing (AM) techniques: (**a**) L-PBF, (**b**) EB-PBF, (**c**) LP-DED, (**d**) AW-DED, (**e**) HLADED [52], (**f**) WAAM [51].

PBF represents a significant branch of AM, whereby the powder dispersed on the substrate is selectively melted through a continuous process of melting and solidification [45]. The most common heat sources employed in PBF are laser (L-PBF) and electron beam (EB-PBF) [8]. The L-PBF operation comprises three principal steps: firstly, the establishment of a CAD model; secondly, the presetting of specific parameters and configuration of the scanning path; and thirdly, the use of a galvanometer-driven laser heat source to fuse the powder under computer control. Following the initial fusion of a single layer, the *Z*-axis is reduced, and the powder is fused in a successive manner. In contrast to L-PBF, EB-PBF employs a pre-laid powder that is melted layer by layer in a vacuum chamber using a focused electron beam. The operation steps are essentially identical to those of L-PBF [46]. The PBF diagram is presented in Figure 9a,b [47]. DED is also one of the important processes for AM, and its basic principle is the same as that of L-PBF. However, the method of powder material deposition is quite different; during the manufacturing process, the powder material is injected through a nozzle into a molten pool formed by heating the substrate by a heat source [48]. The heat source may be a laser, an electron beam, or a plasma arc. Figure 9c–e [49] depicts the diagram of a DED system. DED can be employed to fabricate samples on uneven substrates, although this is not a suitable approach for PBF [12]. WAAM is a critical segment of AM, where an electric or plasma arc is used as a heat source. The metal wire is melted by the discrete stacking principle to form a three-dimensional component on the surface of the substrate [50]. Figure 9f presents a schematic diagram of the WAAM [51].

The layer-by-layer preparation process inherent in AM technology enables the fabrication of complex components. In addition, each AM technology possesses distinctive advantages. Therefore, AM technology is particularly well suited to the fabrication of high-performance materials and the development of new materials [43].

## 4. Research Status of Laser Additive Manufacturing

### 4.1. Powder Bed Fusion

**Figure 10:**
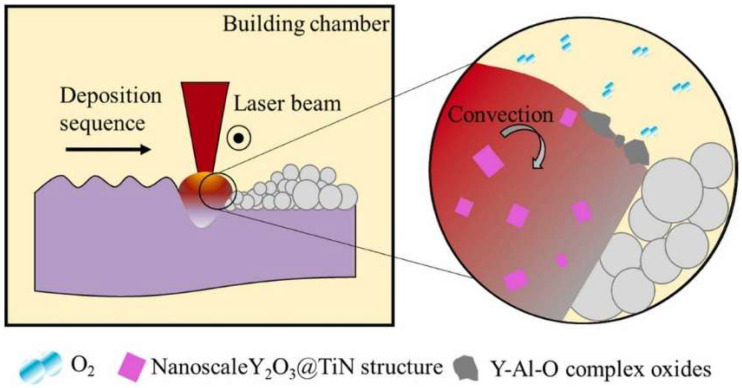
Schematic diagram of the formation process of nanoscale Y_2_O_3_@TiN structure and micro-scale Y-Al-O complex oxides during the PBF-LB process [19].

Yan et al. [53] prepared ODS 316L alloys (ODS-316L) using L-PBF. It was found that the high laser power and rapid solidification characteristics of L-PBF technology can transform oxides into nanoparticles and improve the properties of materials. ODS-IN718 reinforced with Y_2_O_3_ oxide was prepared by Song et al., with the L-PBF approach exhibiting excellent mechanical properties [54]. It was noted that the high-temperature tensile strength of yttrium oxide-reinforced IN718 is about 100 MPa higher than that of the unadded one. Xu et al. [55] employed L-PBF to melt Y-doped NiCrFe powder in order to prepare an ODS alloys and in situ generate Y_2_O_3_ nanoparticles. During the melting process, the yttrium oxide particles situated at the grain boundaries serve as pinning points for the dislocations, thereby markedly enhancing the mechanical properties of ODS NiCrFeY. Wang et al. [19] fabricated an ODS alloy (Fe-22Cr-5.1Al-0.5Ti-0.26Y (wt.%)) using the LPBF technology. The study found that two phases appeared in the ODS alloys: one is the Y-Al-O composite phase formed on the surface of the molten pool; the other is the nano-sized Y_2_O_3_@TiN core–shell structure (Figure 10). Li et al. [56] found that low laser power and high scanning speed could not only increase the density of oxide particles but also reduce the size of oxide particles during the preparation of ODS Fe14Cr alloys by L-PBF. The laser power also dramatically affects the degree of recrystallization observed in the remelted zone, e.g., the <100> fiber texture decreases as the laser power decreases.

**Figure 11:**
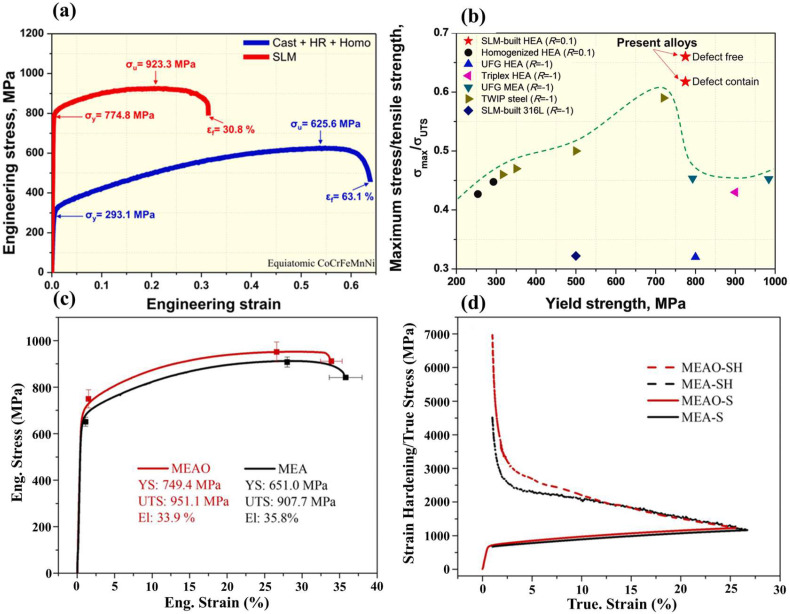
(**a**) Typical tensile stress–strain curves of L-PBF constructed and conventionally processed CoCrFeMnNi high-mixed alloys; (**b**) maximum stress/tensile strength–yield strength plot with other metallic-, medium-, and high-entropy alloys [58]; (**c**) engineering stress–strain curves of the LPBF-built MEA and MEAO samples; (**d**) corresponding true stress–strain (S) curves and the strain hardening (SH) curves [59].

High-entropy alloys (HEAs) are composed of at least five principal elements, with an equal molar ratio or a molar ratio that is close to equal [57]. The differing atomic radii of the constituent elements can give rise to significant lattice distortion, which may impede dislocation movement during deformation. The CoCrFeMnNi high-entropy alloys prepared by L-PBF technology by Kim et al. [58] is a single-phase FCC structure with a uniform composition. The prepared parts exhibited excellent mechanical properties with an elongation and a yield strength of 30.8% and 774.8 MPa, respectively (Figure 11a,b). Furthermore, the heterogeneous grain structure and dislocation network-induced substructure, in conjunction with the high-density in situ formed nanoscale oxides and the deformation twins generated during cyclic loading, collectively contribute to the exceptional fatigue resistance of L-PBF constructed HEAs.

The equimolar CrCoNi is single-phase FCC medium-entropy alloys (MEAs) with excellent properties such as high strength, good plasticity and toughness, radiation resistance, and corrosion resistance [59,60,61]. It was found that the strength and ductility of MEAO improve compared with MEAs samples (Figure 11a,b). Han et al. [59] prepared CrCoNi medium-entropy alloys rich in oxide particles (MEAO) via laser powder bed fusion (L-PBF). It was found that compared with MEAs alloys, MEAO showed improvements in both strength and ductility (Figure 11c,d).

### 4.2. Direct Energy Deposition

In contrast to PBF technology, DED technology enables the preparation of parts on non-flat substrates, such as the repair and remanufacturing of aerospace parts [12]. DED has been employed in several engineering fields, including the fabrication of cladding materials, pipelines, and structural material coatings [13]. Doñate et al. [48] synthesized Y_2_O_3_ nanoparticle colloids by laser fragmentation in liquid and subsequently processed by DED. It is evident that during the DED manufacturing process, the nanoparticles are agglomerated. The size of the nanoinclusions in the DED steel sample is larger, but the content of nanoinclusions in the steel is particularly lower. Li et al. [62] prepared CoCrFeNiMn HEAs samples with nano-sized Y_2_O_3_ particles by DED. The results showed that with the increase of Y_2_O_3_ content, the number of cluster particles used for nucleation increased, which hindered the growth of grains, and thus the material had excellent mechanical properties. In the presence of oxides or nitrides within the alloy powder, oxygen or nitrogen can facilitate the in situ synthesis of a second-phase precipitate [63]. The austenitic stainless steel 316L parts manufactured by AM exhibit high strength and ductility, which is attributed to grain boundary strengthening and the segregation of solute atoms during rapid cooling [64]. Grandhi et al. [65] successfully fabricated an ODS-316L alloys with an austenite FCC microstructure by DED. The incorporation of oxide particles into the alloys results in an increase in hardness and elastic modulus, which are higher than those of pure 316L.

### 4.3. Wire and Arc Additive Manufacturing

**Figure 12:**
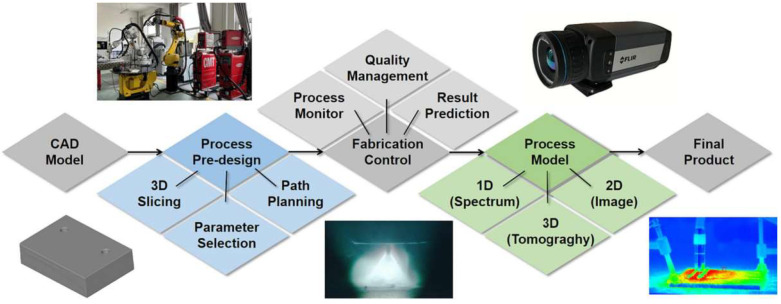
Visualization process of the WAAM process [66].

Figure 12 shows the manufacturing process of WAAM, which possesses the advantages of a high deposition rate, the ability to manufacture complex parts, and cost savings [66,67]. Therefore, it is extensively employed in aerospace [68], architectural [69], oceanographic [70], and other related fields. Y_2_O_3_ dispersion-strengthened low-activation ferrite/martensite (RAFM) steel was prepared by WAAM method by Zhou et al. [71]. Nanoscale Y_2_Ti_2_O_7_ interacted with dislocations and grain boundaries in the steel after irradiation with 2.5 MeV Fe ions at 450 °C. Following irradiation, the lath martensite structure of the ODS-RAFM steel matrix demonstrated stability, exhibiting commendable resistance to radiation-induced damage. Shi et al. [72] investigated modified 9Cr-1Mo ferrite/martensitic steel prepared by WAAM and found that the widths of the martensite laths in hot-rolled steel and WAAM steel are 0.4–0.6 μm and 0.2–0.4 μm, respectively. The finer martensite laths present in WAAM steel result in a higher cyclic stress response. Zhou et al. [73] fabricated thin-walled parts of ODS-9Cr-RAFM steel using WAAM technology, where the flux-cored wire was prepared through the following process: nano-Y_2_O_3_ powder was mixed with Ti-containing 9Cr-RAFM steel powder, the mixture was filled into a Fe-Cr steel tube, and then the assembly was processed into wire via cold drawing. Dong et al. [74] fabricated carbon steel using WAAM and investigated its corrosion performance. The results showed that in a 3.5 wt.% NaCl solution, the WAAM carbon steel exhibited a self-corrosion potential of −0.750 V, a pitting potential of −0.527 V, and a self-corrosion current density of 7.3 × 10^−6^ A/cm^2^. After corrosion, the pearlite structure of the carbon steel could still maintain a relatively intact morphology. Michel et al. [75] found in their study that the rods produced by WAAM only corrode under the harsh condition of 100% relative humidity. Mannan et al. [76] revealed through research that the 316L stainless steel by WAAM has a worm-like δ-ferrite structure. Owing to the fine dendritic structure, which facilitates the outward diffusion of chromium, a thicker chromium oxide layer is formed on the surface of the specimens. Consequently, the 316L alloy exhibits a low corrosion rate in both molten salt and air environments.

In general, scholars have developed effective methods for introducing nano-oxide particles to improve the high-temperature mechanical properties, radiation resistance, and creep properties of ODS alloys. The resulting experimental data provide the possibility for the application of additive manufacturing ODS alloys (AM-ODS) in high-temperature extreme environments such as aerospace and nuclear energy. However, the comprehensive properties of ODS alloy parts fabricated by the above three AM methods are not identical. The PBF process has extremely high solidification rates (L-PBF: 10^4^–10^7^ K/s [77,78]; EB-PBF: 10^4^–10^6^ K/s [79]). Meanwhile, the large temperature gradient generated during solidification induces a strong Marangoni flow effect in the molten pool, which facilitates the formation of nanoparticles smaller than 100 nm in the matrix [80,81]. The fine and dispersedly distributed nano-oxides can achieve grain refinement, thereby reducing the formation of defects such as porosity and cracks [73,82]. Relevant studies have shown that nano-oxide particles in nickel-based materials can reduce the probability of hot crack formation at high-angle grain boundaries [83,84]. When fabricating ODS alloys using the DED and WAAM processes, since the cooling rates of DED and WAAM are much lower than that of the PBF process (L-DED: 10^3^–10^4^ K/s [85]; WAAM: 10^2^–10^3^ K/s [86]), the nano-oxide particles remain in the molten pool for a longer time, leading to a significant agglomeration tendency of the nanoparticles [87]. This phenomenon results in a lower number density and larger size of nanoparticles in the fabricated ODS alloys, which is not conducive to grain refinement and makes the alloys more prone to forming defects such as porosity and cracks. Therefore, PBF is one of the most commonly used processes for fabricating ODS alloys.

## 5. Conclusions

Although AM-ODS alloys possess a multitude of advantageous properties, numerous pressing issues remain to be addressed in the AM process. There are generally two types of raw materials for AM-ODS alloys: powders and wires. The raw material powders must contain nano-sized oxide particles with fine size and uniform distribution, and these oxide particles should maintain a spherical shape as much as possible to ensure good fluidity during the laser heating and melting process. Otherwise, the phenomena of oxide agglomeration and uneven distribution will occur during processing, which, in turn, reduces the performance of the workpiece. The preparation of such high-quality powders not only imposes extremely stringent requirements on process parameters but also involves complex preparation procedures, which significantly restricts their large-scale production and application. An important reason for the relatively limited research on preparing ODS alloys WAAM is the difficulty in manufacturing usable oxide wires [15]. Therefore, exploring powders and oxide wires suitable for AM holds important research value and application prospects [40]. In addition, the method of achieving the in situ synthesis of oxide particles on the substrate surface through chemical reactions is also one of the hot topics in future research [15]. Although ODS alloys prepared by AM exhibit excellent comprehensive properties, the extremely high cooling rate during the AM process leads to high residual stress inside the formed material. Moreover, the non-equilibrium solidification during the cooling process may cause the segregation of elements such as Cr and Mo at grain boundaries, inducing intergranular corrosion [88,89,90,91,92]. Meanwhile, AM-ODS alloy parts may have relatively high surface roughness, which affects the fatigue performance of the parts and limits their applications [93]. Therefore, additional post-processing treatments such as milling, electrochemical polishing, and heat treatment on AM-ODS alloys can significantly improve surface precision and enable the parts to achieve excellent performance [94,95,96,97].

Secondly, the most extensively studied AM-ODS materials are currently Fe-based and Ni-based alloys, while research on ODS-Cu and ODS-Al materials is relatively limited. ODS-Cu combines high thermal conductivity, high plastic toughness, excellent high-temperature stability, and irradiation resistance, showing great application potential in the field of heat sink materials for future magnetic fusion reactors [38]. However, during the preparation of ODS-Cu, oxide particles cannot be uniformly dispersed in the matrix; instead, they tend to accumulate at grain boundary edges, which easily leads to stress concentration and cracking, thereby reducing the strength and ductility of ODS-Cu and its alloys. Al-based materials are lighter than Fe-based and Ni-based materials and possess good machinability. Meanwhile, ODS-Al alloys also exhibit excellent neutron irradiation resistance and corrosion resistance. Nevertheless, for ODS-Al alloys, their high reflectivity and high thermal conductivity can cause the added powders or elements to volatilize easily under laser irradiation, resulting in defects such as cracks. Future researchers are likely to focus on exploring different preparation methods and process optimization approaches to improve the microstructure and mechanical properties of ODS-Cu and ODS-Al alloys.

Finally, since precise control of the process parameters (e.g., laser power, scanning speed, powder coating thickness, scanning strategy, etc.) of the AM-ODS process is required to ensure that the resulting workpieces have excellent mechanical properties, machine learning and artificial intelligence techniques are now also being applied in the field of AM-ODS. Bai et al. [98] selected ODS alloys in the range of 200–300 groups and established the relationship between the ODS alloys in the mathematical model between the key components and the tensile properties of the workpiece, using machine learning algorithms to infer the existence of an optimum value between the tensile strength of the ODS alloys and the content of oxide particles, and listed the composition ratio of the ODS alloys with a room temperature tensile strength of more than 1400 MPa. Therefore, optimizing the process parameters of AM-ODS alloys using new simulation methods and tools may also be one of the future trends of AM-ODS.

## Figures and Tables

**Table 1:** Significant advantages of additive manufacturing technology compared with traditional manufacturing technologies.

Aspects	Advantage
Resolution	Micro-scale (μm) fine feature formation can be achieved.
Material Cost	It features extremely an high material utilization rate and lower comprehensive costs for small-batch and complex components.
Design Limitations	It has no minimum wall thickness limitation, enables free formation of complex internal structures, and breaks the “geometric constraints” of traditional manufacturing.
Processing Speed	Printing can be initiated directly from a digital model, eliminating the time required for mold design, manufacturing, and debugging in traditional manufacturing, thus achieving faster processing speed.

## Data Availability

No new data were created or analyzed in this study. Data sharing is not applicable to this article.
